# Modelling and Design of a Dual Depletion PIN Photodiode as Temperature Sensor

**DOI:** 10.3390/s23104599

**Published:** 2023-05-09

**Authors:** Ricardo A. Marques Lameirinhas, João Paulo N. Torres, Catarina P. Correia V. Bernardo

**Affiliations:** 1 Department of Electrical and Computer Engineering, Instituto Superior Técnico, 1049-001 Lisbon, Portugal; 2 Instituto de Telecomunicações, 1049-001 Lisbon, Portugal; joaotorres@ist.utl.pt; 3 Academia Militar/CINAMIL, Av. Conde Castro Guimarães, 2720-113 Amadora, Portugal

**Keywords:** InP-In_0.53_Ga_0.47_As photodiode, optical sensors, optics, optoelectronics, photodiodes, PIN structures

## Abstract

Nowadays, optical systems play an important role in communications. Dual depletion PIN photodiodes are common devices that can operate in different optical bands, depending on the chosen semiconductors. However, since semiconductor properties vary with the surrounding conditions, some optical devices/systems can act as sensors. In this research work, a numerical model is implemented to analyze the frequency response of this kind of structure. It considers both transit time and capacitive effects, and can be applied to compute photodiode frequency response under nonuniform illumination. The InP-In${}_{0.53}$Ga${}_{0.47}$As photodiode is usually used to convert optical into electrical power at wavelengths around 1300 nm (O-band). This model is implemented considering an input frequency variation of up to 100 GHz. The focus of this research work was essentially the determination of the device’s bandwidth from the computed spectra. This was performed at three different temperatures: 275 K, 300 K, and 325 K. The aim of this research work was to analyze if a InP-In${}_{0.53}$Ga${}_{0.47}$As photodiode can act as a temperature sensor, to detect temperature variations. Furthermore, the device dimensions were optimized, to obtain a temperature sensor. The optimized device, for a 6 V applied voltage and an active area of 500 $\mu m^{2}$, had a total length of 2.536 μm, in which 53.95% corresponded to the absorption region. In these conditions, if the temperature increases 25 K from the room temperature, one should expect a bandwidth increase of 8.374 GHz, and if it decreases 25 K from that reference, the bandwidth should reduce by 3.620 GHz. This temperature sensor could be incorporated in common InP photonic integrated circuits, which are commonly used in telecommunications.

## 1. Introduction

Photodiodes are important components in optical systems. They allow us to convert optical into electrical power and, consequently, the transition between the two domains allow us to consider both domains’ advantages when designing a communication system. A commonly analyzed structure is the PIN type, mainly due to its good frequency response, great sensitivity, and excellent signal-to-noise noise ratio [1,2,3,4,5,6,7].

Other figures of merit are also important to consider. The most important figures of merit related to photodetection are responsivity and photoconductive gain; efficiency and quantum efficiency; spectral selectivity and ultraviolet-to-visible rejection ratio; detectivity and noise-equivalent power; and bandwidth and time response. Different photodetectors can be compared using quantitative results from this list. In optical communications, bandwidth and quantum efficiency are important figures of merit, mainly because bandwidth is related to the system bitrate and the quantum efficiency is related to the photodetector sensitivity [1,2,5].

Furthermore, a given photodetector might act as a sensor, due to the fact that the material properties must change with certain stimuli [7]. This might lead to changes in the figure of merit values. Thus, it is possible to optimize the function of a photodiode to monitor certain stimuli, adding further capabilities to optical communication systems.

The use of these periodic structures as sensors has been studied, since their manufacture and reproducibility seems not to be a difficult issue for specialized companies [1,5,8]. The main focus is on the optimization process, since the design goals are different. The research problem is now more complex. Besides the optimization of the device dimensions to reach a certain figure of merit and client specifications, to design a sensor, it is necessary to analyze it with different stimuli and verify the variations of the device response.

In the case of semiconductors materials, some material dependencies are commonly studied, such as the variation of their properties with temperature [7]. InP-In${}_{0.53}$Ga${}_{0.47}$As photodetectors work at 1300 nm, with tens or hundreds of GHz of bandwidth [5,8,9,10]. This temperature sensor could be incorporated into common InP photonic integrated circuits, which have a excellent cost–performance relation. InP photonic integrated circuits are commonly used in telecommunications. The main aim of this research work was to optimize a dual depletion InP-In${}_{0.53}$Ga${}_{0.47}$As PIN photodiode as a temperature sensor, exploring its bandwidth variations. In order to achieve this, a model based on continuity equations was implemented, exploiting matrix relations to determine the frequency response of this type of device.

## 2. Methodology

### 2.1. Structural Analysis

**Figure 1:**
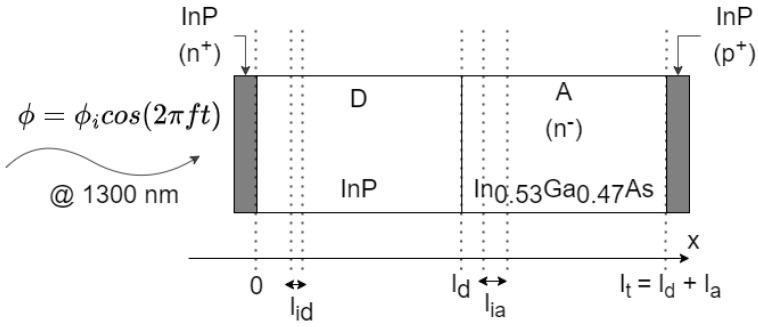
InP-In${}_{0.53}$Ga${}_{0.47}$As PIN photodiode schematic.

The analyzed structure was a dual depletion PIN photodiode, as illustrated in Figure 1. It is composed of two InP contacts, one n+ where the light flux is incident on, and another p+ on the other side. Between them, there are two regions. After the n+ contact, there is a drift InP region (D) and an absorption In${}_{0.53}$Ga${}_{0.47}$As region (A). The contact dimensions and interfaces are neglected, and the devices have a total $l_{t}$ length, divided into $l_{d}$ and $l_{a}$ for the drift and absorption region lengths, respectively. In addition, $p_{a}$ is defined as the absorption layer thickness percentage of the total device, $l_{a}/l_{t}$. These semiconductors were chosen in order to have a high absorption between 1.0 μm and 1.6 μm, namely at 1.3 μm in the center of the original optical band (O-band). Both semiconductors were lattice matched [1,4,7,8,10].

Different responses can be obtained if radiation is incident on either side; for instance, on the p contact side or directly on the intrinsic region.

A model based on the capacitive and transit time effects of a dual depletion PIN photodiode was implemented, which was previously proposed and validated in [1]. The dual depletion PIN photodiode capacitive effects are mainly due to its junction capacitance and certain other parasitic effects, such as those related to the pads and packages. On the other hand, the transit time effects are associated with the electron and hole transport in the drift and absorption regions, due to the electric field. Consequently, they are related to the carrier’s drift velocity and the length of both regions [1,8,9,10].

Thus, both effects should be analyzed in order to evaluate a device’s performance. It is known that the longer the device length, the shorter the transit time will be. On the other hand, by increasing the device length, the capacitance will increase. For this reason, the photoconversion of a device is dependent on the device geometry [1,8,9,10].

### 2.2. Proposed Method

Based on continuity equations, it is possible to establish matrix relations to obtain the frequency response of this kind of device. The analytical solution of these equations can be obtained for constant electrical fields [1]. However, the used methodology decomposes the regions into several layers of a constant electric field, but this can change among them. Analytical solutions can be obtained in each layer, and by combining the solution coefficients, it is possible to compute the frequency response. Consequently, the higher the number of layers, the higher the accuracy of the numerical method.

In the analyzed PIN structure, the electric field is constant in the drift region and it varies linearly in the absorption region. In the frequency domain expressions, (1) and (2) are used to compute the current of the electron, $J_{in}$, and holes, $J_{ip}$, respectively, in the i-th layer of the constant electric field, considering the respective drift velocities $\nu_{in}$ and $\nu_{ip}$ [1].

(1) $$\begin{array}{c} \frac{j\omega }{\nu_{in}}J_{in}\left(x,\omega \right)=\frac{d\;J_{in}\left(x,\omega \right)}{d\;x}+G_{i}\left(x,\omega \right) \end{array}$$

(2) $$\begin{array}{c} \frac{j\omega }{\nu_{ip}}J_{ip}\left(x,\omega \right)=\frac{d\;J_{ip}\left(x,\omega \right)}{d\;x}+G_{i}\left(x,\omega \right) \end{array}$$

The PIN structure was illuminated on the n+ side contact using a sinusoidal optical flux of amplitude $\phi_{i}$ and frequency ω, leading to an electron–hole pair generation rate $G_{i}\left(x,\omega \right)$, defined in expression (3) [1]. This had a null value in the drift region and was dependent on the absorption coefficient α and on the electron charge module *q* in the absorption region. The optical stimuli was absorbed in this region. The *x* coordinate was substituted by the value of $x_{i}$ associated with each layer. Once again, the accuracy of the numerical method was increased by increasing the number of layers.

In contrast to the previous study [1], where this method was proposed, in this article, the null branch of $G_{i}\left(x,\omega \right)$ was not used. To improve the method’s accuracy, both drift and absorption regions were characterized using the complex electrical permittivity or equivalently using the complex refractive index. Consequently, the drift region was characterized identically to the absorption region, but with different values.

(3) $$G_{i}\left(x,\omega \right)=\{\begin{array}{c} 0\;\;\;\;\;\;\;(0\leq x\leq l_{d})\\ q\;\alpha \;\phi_{i}\;e^{-\alpha (x-l_{d})}\;\;\;\;\;\;\;\left(l_{d}\leq x\leq l_{d}+l_{a}\right) \end{array}$$

The linear coefficients of $T_{i}$, $S_{i}$, $R_{i}$, and $D_{i}$ for each i-th layer were obtained by solving the continuity equations [1].

Expression (4) relates $T_{i}$ and $S_{i}$ coefficients, defined in expressions (5) and (6), with the electron and hole current densities. $T_{i}$ are named as current transfer matrices and $S_{i}$ are the current contribution from the optical sources.

(4) $$\left[\begin{array}{c} J_{p}\left(x_{i}\right)\\ J_{n}\left(x_{i}\right) \end{array}\right]=T_{i}\left[\begin{array}{c} J_{p}\left(x_{i-1}\right)\\ J_{n}\left(x_{i-1}\right) \end{array}\right]+S_{i}$$

(5) $$T_{i}=\left[\begin{array}{cc} T_{ipp} & T_{ipn}\\ T_{inp} & T_{inn} \end{array}\right]$$

(6) $$S_{i}=\left[\begin{array}{c} S_{ip}\\ S_{in} \end{array}\right]$$

On the other hand, $R_{i}$ and $D_{i}$ are settled in expression (7), where $p_{i}\left(\omega \right)$ is the partial electrode current [1].

On the other hand, $R_{i}$ and $D_{i}$ are settled in expression (7), where $p_{i}\left(\omega \right)$ is the partial electrode current. $D_{i}$ is scalar and it is related to optical sources, whereas $R_{i}$ is defined in (8) and it relates the left-hand terminal currents and $p_{i}\left(\omega \right)$.

(7) $$p_{i}\left(\omega \right)=\int_{l_{i}}\left[J_{n}\left(x,\omega \right)+J_{p}\left(x,\omega \right)\right]d\;x=S_{i}^{T}\left[\begin{array}{c} J_{p}\left(x_{i-1}\right)\\ J_{n}\left(x_{i-1}\right) \end{array}\right]+D_{i}$$

(8) $$R_{i}=\left[\begin{array}{c} R_{i_{p}}\\ R_{i_{n}} \end{array}\right]$$

The multilayer structure coefficients were obtained by recursively computing the expression set (9). These operations allowed the union of the i-th and the (i+1)-th layer, its coefficients being defined as (i+1,i). At the end of this computation, the coefficients were representative of the entire structure’s performance.

(9) $$\left\{ \begin{array}{c} T_{i+1,i}=T_{i+1}T_{i}\\ S_{i+1,i}=S_{i+1}+T_{i+1}S_{i}\\ {R_{i+1,i}}^{T}={R_{i+1}}^{T}T_{i}+R_{i}^{T}\\ D_{i+1,i}={R_{i+1}}^{T}S_{i}+D_{i+1}+D_{i} \end{array}\right.$$

The frequency response of the photogenerated current I(ω) is defined by expressions (10) and (11), related to the whole structure coefficients.

(10) $$I\left(\omega \right)=\frac{\delta (\omega )}{l_{a}+l_{d}}$$

(11) $$\delta \left(\omega \right)=D-\frac{R_{n}S_{n}}{T_{nn}}$$

The coefficients $T_{i}$ and $S_{i}$ were deduced in [1] as presented in expressions (12) and (13), as well as the coefficients of $R_{i}$ and $D_{i}$ in expressions (14) and (15). $f\left(\theta \right)=\frac{1-e^{-\theta }}{\theta }$ is denoted as an auxiliary function, to simplify the expressions’ presentation, as well as $\tau_{i_{n}}=\frac{l_{i}}{\nu_{i_{n}}}$ and $\tau_{i_{p}}=\frac{l_{i}}{\nu_{i_{p}}}$ as the electron and hole transit time in the i-th layer, respectively.

(12) $$T_{i}=\left[\begin{array}{cc} e^{-j\omega \tau_{i_{p}}} & 0\\ 0 & e^{j\omega \tau_{i_{n}}} \end{array}\right]$$

(13) $$S_{i}=q\alpha \phi_{i}l_{i}e^{-\alpha x_{i}}\left[\begin{array}{c} f(j\omega \tau_{i_{p}}-\alpha l_{i})\\ -f(-j\omega \tau_{i_{n}}-\alpha l_{i}) \end{array}\right]$$

(14) $$R_{i}=l_{i}\left[\begin{array}{c} f(j\omega \tau_{i_{p}})\\ f(-j\omega \tau_{i_{n}}) \end{array}\right]$$

(15) $$\begin{array}{c} D_{i}=q\alpha \phi_{i}l_{i}^{2}e^{\alpha (l_{i}-x_{i})}\times \\ \left(\frac{f\left(\alpha l_{i}\right)-f\left(-j\omega \tau_{i_{n}}\right)}{\alpha l_{i}+j\omega \tau_{i_{n}}}-\frac{f\left(\alpha l_{i}\right)-f\left(j\omega \tau_{i_{p}}\right)}{\alpha l_{i}-j\omega \tau_{i_{p}}}\right) \end{array}$$

The implemented model is a more general approach in comparison with the one presented in [1]. In this research work, the model was implemented in Python. In this case, the drift and absorption layers were treated equally. In [1], the coefficients for the drift layers were different from the ones for the absorption layer, due to the fact that the absorption coefficient and the carrier velocities must be null. In this article, general expressions are deduced, and every i-th layer (in drift and absorption regions) is considered. This was used to analyze a device with a drift and an absorption region; however, following this novel approach, a N-region structure might be considered, even though it does not characterize the layers as drift and absorption, and only characterizing with the properties of the medium.

In addition, the aim in [1] was to obtain the frequency response of InP-In${}_{0.53}$Ga${}_{0.47}$As photodiodes, to be used in communication systems, whereas in this article the aim was to investigate the use of these photodiodes as temperature sensors, and optimizing their dimensions for that purpose.

### 2.3. Semiconductor Properties

The semiconductors models for the InP drift region and for the In${}_{0.53}$Ga${}_{0.47}$As absorption region at 300 K were obtained from [11,12], respectively. In Table 1 are the parameters used from these references. Moreover, there are the parameters at two other temperatures used in the temperature sensor simulation. The semiconductor property models were introduced into the general model, in order to consider temperature as an initial condition.

The parameter variations with temperature are reported in [7,13,14]. The used carrier velocity models are also dependent on the applied electric field (applied voltage) and on the residual donor concentration in the absorption region [1].

A small signal equivalent circuit was used to convert the optical excitation into an electrical current, as illustrated in [1]. In this computation, an infinity leakage resistance was assumed, in order to obtain a reasonable transfer function. Expression (16) was deduced from the equivalent circuit, relating the output current $I_{0}$ as a function of the optical excitation, $I_{0}=H\times I$, for each working frequency [1,2].

(16) $$H=\frac{1}{-\omega^{2}R_{L}R_{s}C_{p}C_{1}+j\omega \left(C_{1}\left(R_{L}+R_{s}\right)+R_{L}C_{p}\right)+1}$$

Moreover, similar to what was reported in [1], a donor concentration of $N=10\times 10^{21}$ m${}^{-3}$, a parasitic capacitance of $13\times 10^{-15}\;F$, and a load resistance of 50 Ω were assumed.

In this research work, the number of absorption layers was set to 40 and there was only one drift layer. However, the method was implemented considering them as variables. The variation in the drift layer number may be useful if the drift region is absorption, which was not the case.

## 3. Method Validation

Before optimizing the frequency response of the dual depletion InGaAs/InP PIN photodiode, the implemented approach was validated with the prior one, confirming that its execution was correct.

**Figure 2:**
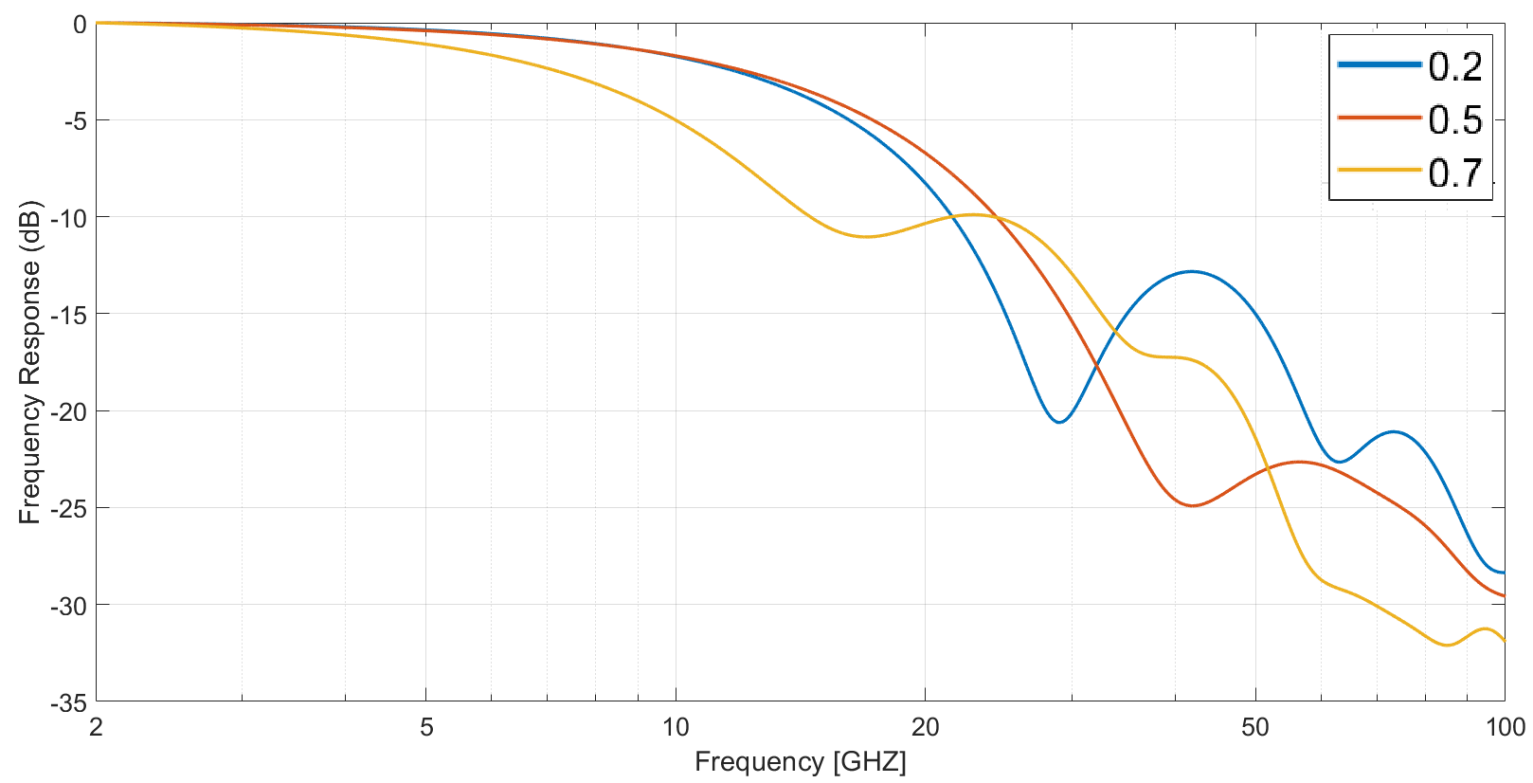
Frequency response of a dual depletion In${}_{0.53}$Ga${}_{0.47}$As PIN photodiode with an active area of 500 $\mu m^{2}$ and a total length of 3 μm, working with a 10 V applied voltage, as a function of its absorption layer percentage at room temperature.

Thus, for $l_{t}$ = 3 μm and a 10 V applied voltage, the obtained frequency response is presented in Figure 2, for three different values of $p_{a}$ and considering an active area of 500 $\mu m^{2}$. In the design stage, by sweeping the absorption layer thickness percentage it was possible to tune the device’s optical response, namely its bandwidth. In these conditions, it was also noticeable that this thickness variation could lead to significant fluctuations in the response at high frequencies. However, these devices in these conditions have a low-pass characteristic.

**Figure 3:**
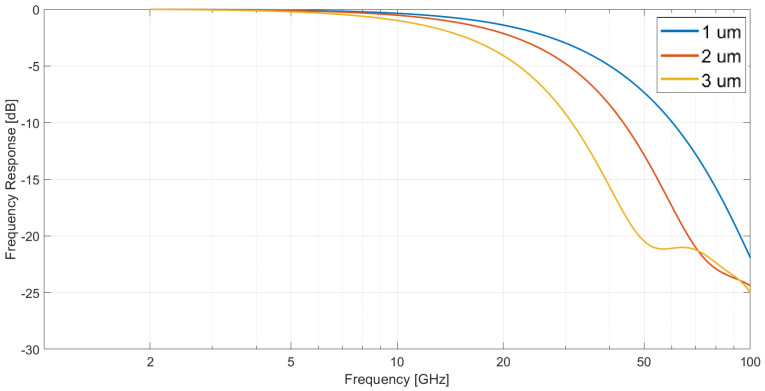
Frequency response of a dual depletion In${}_{0.53}$Ga${}_{0.47}$As PIN photodiode with an active area of 500 $\mu m^{2}$ and an absorption layer percentage of 50%, working with a 10 V applied voltage, as a function of its total length at room temperature.

On the other hand, keeping the absorption layer percentage at 0.5=50%, but varying its thickness and the total one, it was concluded that the lower the total length, the better the response seemed to be, since it was smoother and it had no peaks in the analyzed frequency range. In Figure 3, these results are shown, assuming a 10 V applied voltage.

In [1,2,3], other interesting results are reported, applying different voltages and device dimensions. However, there was no optimization process and the device was not a sensor but a photodiode in a communication system. In this article, the optimization process was performed in a temperature sensor, based on the variation in semiconductor properties with it.

## 4. Influence of the Structural Dimensions on the Bandwidth

This research work aimed to explore the possibility of using a dual depletion In${}_{0.53}$Ga${}_{0.47}$As PIN photodiode as a temperature sensor. Since it was intended to verify how the photodiode bandwidth changes with temperature, it was necessary to have more details about how the response varies with the photodiode dimensions.

Various photodiode responses for an active area of 500 $\mu m^{2}$ and a 6 V applied voltage were determined, by varying $l_{t}$ and $p_{a}$. The total length $l_{t}$ was in a range between 0.05 μm and 3 μm; and, on the other hand, $p_{a}$ varied from 0 to 1. Both varied linearly, with a total of 50 equidistant points, and all their combinations were considered.

**Figure 4:**
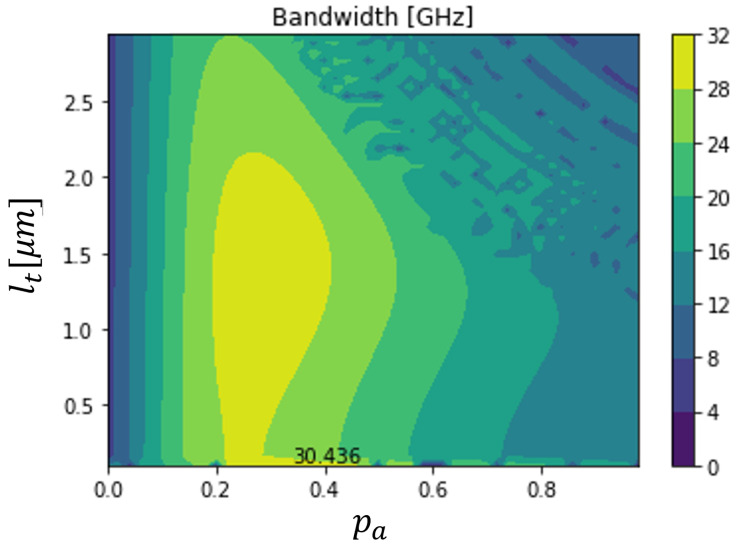
Bandwidth of a 500 $\mu m^{2}$ dual depletion In${}_{0.53}$Ga${}_{0.47}$As PIN photodiode, working with a 6 V applied voltage, as a function of its total length and its absorption layer percentage at room temperature.

Figure 4 shows a contour plot, where the device dimensions are varied. This problem was a multi-parameter optimization. For each combination, the device bandwidth was determined and a graph was plot. Based on this kind of figure, it was possible to verify the best dimension combinations and determine the influence of each parameter on the frequency response.

The bandwidth maxima was around 30.436 GHz, with this value being within the expected range [1,9,10,13]. In this case, it was possible to conclude that the maximum bandwidth was higher when the absorption layer percentage was around 20% to 45% percent of the total device length. In addition, the maximum bandwidth was obtained for shorter devices, but it was also possible to obtain quite similar devices with moderate dimensions.

## 5. Temperature Sensor

The temperature sensor was simulated using a reference room temperature (300 K) and variations of 25 K (or 25 ${}^{\circ }$C). The parameters at different analyzed temperatures are presented in Table 1. The semiconductor properties varied with temperature, a phenomena modeled using previously validated approaches from other authors, as presented in references [7,13,14].

Using this approach, one can analyze the best photodiode dimensions for obtaining the highest bandwidth variation. Thus, it is possible to obtain the temperature from this analysis. First, it is possible to perform spectral analysis, and by determining the bandwidth, estimate the temperature or, at least, verify if the temperature has varied within a certain range (in this case, verify if it was 25 K). On the other hand, it is possible to use only a certain pulse frequency. By analyzing the output power, the temperature can be determined. If that frequency is higher than the bandwidth, the output power will be at least half of the maximum power. Then, a reference (for instance, the room temperature) should be stored, and the temperature variation can be detected by the output power that is lost.

**Figure 5:**
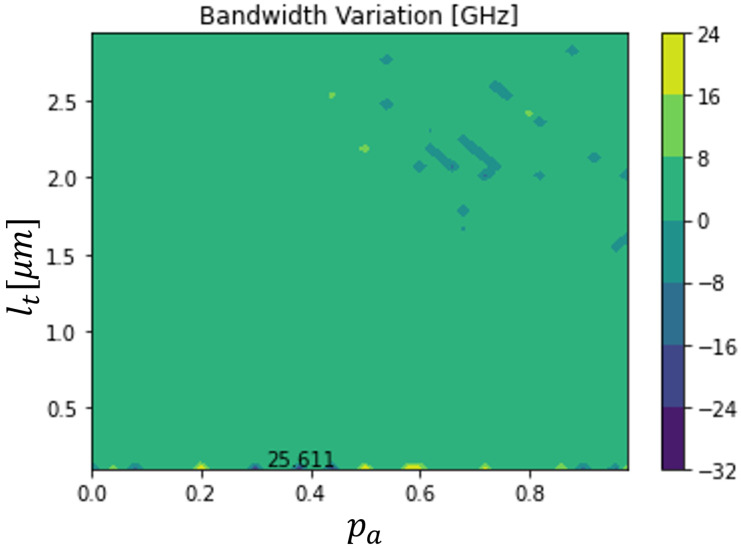
Bandwidth variation of a 500 $\mu m^{2}$ dual depletion In${}_{0.53}$Ga${}_{0.47}$As PIN photodiode, working with a 6 V applied voltage, as a function of its total length and its absorption layer percentage for a variation of −25 K.

**Figure 6:**
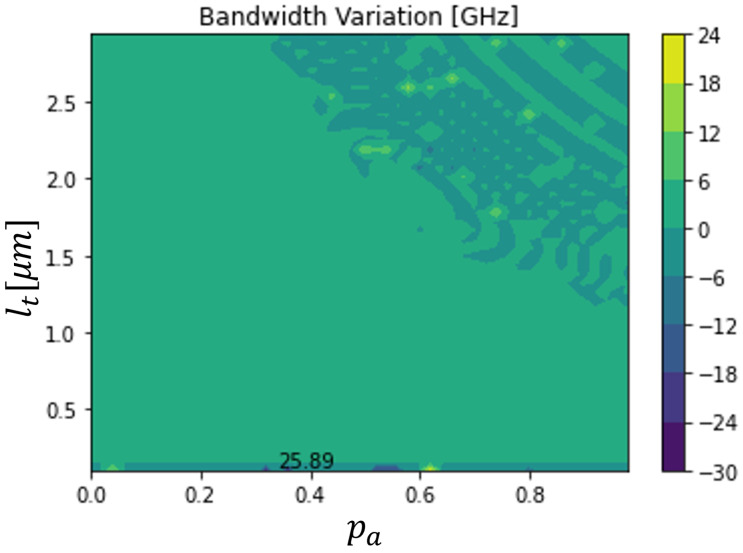
Bandwidth variation of a 500 $\mu m^{2}$ dual depletion In${}_{0.53}$Ga${}_{0.47}$As PIN photodiode, working with a 6 V applied voltage, as a function of its total length and its absorption layer percentage for a variation of +25 K.

Figure 5 and Figure 6 illustrate a rigorous sweep of both the absorption layer percentage ($p_{a}$) and total length ($l_{t}$), for a 10 V applied voltage on a 500 $\mu m^{2}$ device. These figures allowed us to optimize the device, to verify which dimensions gave more sensitivity to temperature variations. It was concluded that there are regions where the bandwidth varies sufficiently to detect the variation, namely with absolute variations higher than 20 GHz.

However, to detect the increase and decrease of temperature, referenced to the room temperature, the bandwidth variations have to have different signs. For that reason, the optimized temperature sensor was not the one shown in Figure 5 and Figure 6. The maximum bandwidth variations in those conditions were −3.620 GHz and 8.374 GHz, respectively, for the temperature decrease and increase, with 10.330 GHz being the reference bandwidth. The optimized device had a total length of 2.536 μm and its absorption area was 53.95% of the total length. The obtained bandwidth variations were smaller than the maxima only for an increase or a decrease in temperature in Figure 5 and Figure 6. However, they were sufficiently high to be detected.

## 6. Conclusions

The aim of this research work was to implement a method capable of modeling the frequency response of dual depletion PIN photodiodes. In addition, the device was tested as a temperature sensor, using the same approach to optimization.

The model was validated with previously published results. An In${}_{0.53}$Ga${}_{0.47}$As was analyzed within its absorption region, namely at 1300 nm and at room temperature. The frequency response was obtained in a range from 0 to 100 GHz. The device dimensions were swept and the bandwidth was obtained at the half-power point. Then, it was possible to analyze how these parameters influenced the device’s bandwidth, which was revealed to be a multiparameter optimization problem.

The semiconductors’ properties were studied, in order to investigate the influence of temperature on them. To test the photodiode as a temperature sensor, two other temperature values were analyzed. By varying the semiconductor properties, the influence of temperature on the overall device response was analyzed. Bandwidths were also computed at these temperatures, which were established as 25 K variations from room temperature. By subtracting the bandwidth values at different temperatures, we could verify how this figure of merit was influenced by the temperature.

A sensor was obtained by establishing room temperature as the reference and by choosing a certain pulse frequency. For that frequency, if the output power varied −3 dB, the temperature variation was higher than the 25 K.

The device optimized for a 6 V applied voltage and an active area of 500 $\mu m^{2}$ had a total length of 2.536 μm, in which 53.95% corresponded to the absorption region. In these conditions, if the temperature increases 25 K from the room temperature, one should expect a bandwidth increase of 8.374 GHz, and if it decreases 25 K from that reference, the bandwidth should reduce by 3.620 GHz. Then, by choosing two different frequencies, it would be possible to use this device as a temperature sensor. In this case, 6.711 GHz (obtained bandwidth for 275 K) and 18.704 GHz (obtained for 325 K) should be considered.

## Figures and Tables

**Table 1:** Material parameters at different temperatures (* $1\times 10^{-100}$ was used to emulate zero, in order to test the generalization of the matrix formalism).

		Temperature					
		300 K		275 K		325 K	
**Parameters**	**Units**	**In${}_{0.53}$Ga${}_{0.47}$As**	InP	**In${}_{0.53}$Ga${}_{0.47}$As**	InP	**In${}_{0.53}$Ga${}_{0.47}$As**	InP
Absorption Coefficient (α@1.3 μm)	m${}^{-1}$	$1.15\times 10^{6}$	*	$1.13\times 10^{6}$	*	$1.12\times 10^{6}$	*
Electron Saturation Velocity ($\nu_{nl}$)	m/s	$6\times 10^{4}$	$8.11\times 10^{4}$	$6.2425\times 10^{4}$	$8.11\times 10^{4}$	$5.9775\times 10^{4}$	$8.11\times 10^{4}$
Hole Saturation Velocity (νpl)	m/s	$4.8\times 10^{4}$	$8.11\times 10^{4}$	$5.1325\times 10^{4}$	$8.11\times 10^{4}$	$4.8675\times 10^{4}$	$8.11\times 10^{4}$
Electron Mobility ($\mu_{n}$)	m${}^{2}$V${}^{-1}$s${}^{-1}$	1.05	*	1.15	*	0.969	*
Hole Mobility ($\mu_{n}$)	m${}^{2}$V${}^{-1}$s${}^{-1}$	0.042	*	0.046	*	0.038	*
Electric Permittivity (ϵ)	F/m	$14.1\epsilon_{0}$	$12.56\epsilon_{0}$	$14.1\epsilon_{0}$	$12.56\epsilon_{0}$	$14.1\epsilon_{0}$	$12.56\epsilon_{0}$

## Data Availability

Not applicable.
